# Hyperlactatemia and the Outcome of Type 2 Diabetic Patients Suffering Acute Myocardial Infarction

**DOI:** 10.1155/2016/6901345

**Published:** 2016-11-16

**Authors:** Jovanovic Aleksandar, Peric Vladan, Snezana Markovic-Jovanovic, Radojica Stolic, Jadranka Mitic, Tanja Smilic

**Affiliations:** ^1^Department of Endocrinology, Medical Faculty, University of Pristina, Mitrovica, Serbia; ^2^Department of Cardiology, Medical Faculty, University of Pristina, Mitrovica, Serbia; ^3^Department of Pediatrics, Medical Faculty, University of Pristina, Mitrovica, Serbia; ^4^Department of Urology/Nephrology, Medical Faculty, University of Kragujevac, Kragujevac, Serbia

## Abstract

*Background.* Increased lactate production is frequent in unregulated/complicated diabetes mellitus.* Methods.* Three groups, each consisting of 40 patients (type 2 diabetics with myocardial infarction, DM+AMI, nondiabetics suffering myocardial infarction, MI, and diabetics with no apparent cardiovascular pathology, DM group), were tested for pH, serum bicarbonate and electrolytes, blood lactate, and CK-MB.* Results*. Blood lactate levels were markedly higher in AMI+DM compared to MI group (4.54 ± 1.44 versus 3.19 ± 1.005 mmol/L, *p* < 0.05); they correlated with the incidence of heart failure (*ρ* = 0.66), cardiac rhythm disorders (*ρ* = 0.54), oxygen saturation (*ρ* = 0.72), CK-MB levels (*ρ* = 0.62), and poor short-term outcome. Lactic acidosis in DM+AMI group was not always related to lethal outcome.* Discussion*. The lactate cutoff value associated with grave prognosis depends on the specific disease. While some authors proposed cutoff values ranging from 0.76 to 4 mmol/L, others argued that only occurrence of lactic acidosis may be truly predictive of lethal outcome.* Conclusion*. Both defective glucose metabolism and low tissue oxygenation may contribute to the lactate production in diabetic patients with acute myocardial infarction; high lactate levels indicate increased risk for poor outcome in this population comparing to nondiabetic patients. The rise in blood lactate concentration in diabetics with AMI was associated with increased incidence of heart failure, severe arrhythmias, cardiogenic shock, and high mortality rate.

## 1. Background

Metabolic disturbances may contribute to poor outcome after acute myocardial infarction (AMI) in diabetic patients [1]. Increased lactate production was noted in unregulated diabetes, especially with the occurrence of hyperosmolar hyperglycemic state. Arterial pH usually does not fall below 7.3, but occasionally mild metabolic acidosis may develop due to lactate accumulation. In addition, approximately, 50% of the patients with hyperosmolar hyperglycemic state may have an increased anion gap metabolic acidosis because of increased serum lactate level. Serum lactate may also indicate type 1 lactic acidosis related to sepsis or cardiogenic shock [2] in diabetic patients.

The normal lactate range falls between 0.5 and 1 mmol/L. Hyperlactatemia is mild to moderate increase in blood lactate concentration (2–4 mmol/L) without apparent metabolic acidosis [3]. Lactic acidosis is the type of metabolic acidosis characterized by lactate levels >5 mmol/L [4].

Hyperlactatemia can occur in the setting of adequate tissue perfusion, intact buffering systems, and adequate tissue oxygenation. Lactic acidosis, on the other hand, may also be associated with major metabolic dysregulation, tissue hypoperfusion, the effects of certain drugs or toxins, congenital abnormalities in carbohydrate metabolism, or a markedly increased transient metabolic demand [5, 6].

Type 2 diabetic patients often exhibit hyperlactatemia in association with a reduced aerobic oxidative capacity and a restricted lactate transport. Studies suggest a link between increased lactate levels and the manifestation and progression of insulin resistance. However, the specificities of molecular mechanisms remain unclear, and it is not clear whether elevated lactate levels are a cause or consequence of type 2 diabetes [7].

Hyperlactatemia is commonly used as a diagnostic and prognostic tool in intensive care settings. However, the prognostic value of lactate levels in diabetic patients with acute cardiac conditions is not yet clear.

## 2. Methods

The prospective study was carried out in the period 2006–2008 at Clinical Hospital Centre Pristina, Kosovo, Serbia, encompassing patients at the Department of Cardiology, Coronary Unit, and the Department of Endocrinology. Except for the application of exclusion criteria (see below), the patients were selected randomly, as they were admitted to the hospital during the study period.

### 2.1. Design

Three groups, each consisting of 40 patients, were formed according to the basic pathology. The first group included patients with acute myocardial infarction and confirmed type 2 diabetes; their treatment included lifestyle modifications, oral hypoglycemic agents, and insulin; however, patients receiving biguanides were not included in the study. This was possible since metformin has not yet been accepted as the first choice therapy of type 2 diabetes in our country at the time the research was carried out. Patients with serious renal impairment and severe hepatic disorders, as well as obstructive pulmonary disease, were not included in the analysis, once these pathologies were confirmed by the clinical examination and/or laboratory testing.

The second group consisted of patients suffering acute myocardial infarction with normal blood/plasma glucose and no known history of diabetes. Patients with renal, hepatic, and obstructive pulmonary disorders were excluded from the study. No patients in this group received metformin for the treatment of metabolic syndrome.

The third group included 40 patients with known type 2 diabetes without the underlying cardiovascular pathology. Most of these patients were admitted to hospital for additional treatment options for the inadequate metabolic regulation. This group is a control group in regard to acid-base status and blood lactate levels in diabetes mellitus.

### 2.2. Clinical Methodology

Diabetes was diagnosed as fasting plasma glucose levels ≥7 mmol/L or fasting whole blood glucose ≥6.2 mmol/L in more than one occasion. Since stress and contraregulatory hormones secretion in acute myocardial infarction might lead to false-positive diagnosis, only patients with positive history of diabetes and/or hemoglobin A1c > 6.5% were considered eligible in the group with diabetic and acute myocardial infarction. For the same reason, only individuals with acute myocardial infarction and normal glucose readings and no positive history of diabetes were considered nondiabetics with acute myocardial infarction. All diabetic patients in this group had a history of type 2 diabetes, but those diagnoses relied mainly on approximate clinical criteria such as age, history of diabetes progression, or history of antidiabetic medications treatment; only a few patients had diagnosis based on autoantibody determination, and those belonged to the group of type 2 diabetic patients without apparent cardiovascular pathology. Therefore, some (small number) of patients with delayed-onset or slowly progressive type 1 diabetes could not be excluded with absolute certainty.

Myocardial infarction was diagnosed according to standard diagnostic procedure in the intensive care settings and the diagnosis relied on the serial and/or permanent ECG readings, CK-MB fraction, and troponin laboratory tests, followed by heart ultrasound imaging and measurements. All of the patients with acute myocardial infarction included in the study had STEMI type of AMI. The samples for pH, blood gases, and blood lactate, as well as other routine analyses, were taken on the admission to the hospital/coronary unit; in the moment of taking samples all of the patients were treated with the initial conservative therapy only (before the percutaneous coronary interventions were applied).

Patients with stage 4 or 5 of renal failure (GFR < 30 mL/min/1.73 m^2^), symptomatic hepatic cirrhosis (stage 2 and 3 cirrhosis), respiratory failure due to acute or chronic pulmonary diseases, and neuromuscular disorders as well as diarrheas and malabsorption were excluded from the study. The exclusion of the additional cardiovascular pathology (except for the mild to moderate hypertension) was based on patients' history, physical examination, and ECG readings. The exclusion of renal pathology was based on blood urea and creatinine measurements and GFR calculations and single-sample and 24 h urine analyses; patients with isolated uncomplicated microalbuminuria were not excluded from the study, though.

### 2.3. Laboratory Methods

Samples for acid-base status determination were obtained after the admission and initial diagnosis from capillary blood and analyzed using ABL-500, Renz. Data required for the correction of acid-base parameters—patients' age, weight and height, and body temperature—were obtained within the standard clinical procedure on admission and patients' history. Plasma glucose, urea, serum creatinine LDH, and CK-MB, as well as urinary protein excretion, were determined on ADVIA 1650, Siemens-Bayer. Lactate levels were analyzed from venous blood, using Boehringer's PAP-lactate reagent; blood ketones were determined using Freestyle Optium Neo analyzer and Freestyle Optium blood ketone sticks.

Statistical analyses included Student's* t*-test for small independent samples, analysis of variance, *χ* ^2^ test, and Spearman's rank correlation. The estimation of power for comparing two means was 0.86 for the expected blood lactate and 1.0 for the expected H^+^ concentrations (denoting alpha values 0.05).

The study was carried out at the Department of Cardiology and Department of Endocrinology, Clinical Centre Pristina, University of Pristina, Kosovo. The research was approved by the Ethical Committee of the Medical Faculty, University of Pristina.

## 3. Results

The mean age in three groups of hospitalized patients (patients with diabetes and acute myocardial infarction, DM+AMI, and nondiabetics suffering acute myocardial infarction, AMI and diabetics with no cardiovascular pathology, resp.) was similar, with no significant differences (*p* > 0.05, Table 1). The relatively younger age of the patients (58.2 ± 11.7 and 59.9 ± 13.2, resp.) reflects the fact that many of the older patients were eliminated from the study due to apparent comorbidities or to age-related incidences of diagnostic clues (e.g., fall in glomerular filtration rate, rise in gamma-glutamyl transferase or TSH levels, and chest X-ray showing emphysema) pointing out at potential subclinical or silent conditions that could influence lactate levels.

The males were highly prevalent in groups of patients suffering myocardial infarction (from 65% in DM+AMI group to as high as 82.5% in AMI group) in contrast with normal gender distribution in patients without cardiovascular pathology. All three groups had similar mean BMI (*p* > 0.05, Table 1), as well as the prevalence of hypertensive vascular disease. Cardiac decompensation following MI and hearth rhythm disorders were far more prevalent (*p* < 0.001) in diabetics with AMI comparing to nondiabetics (Table 1).

**Figure 1:**
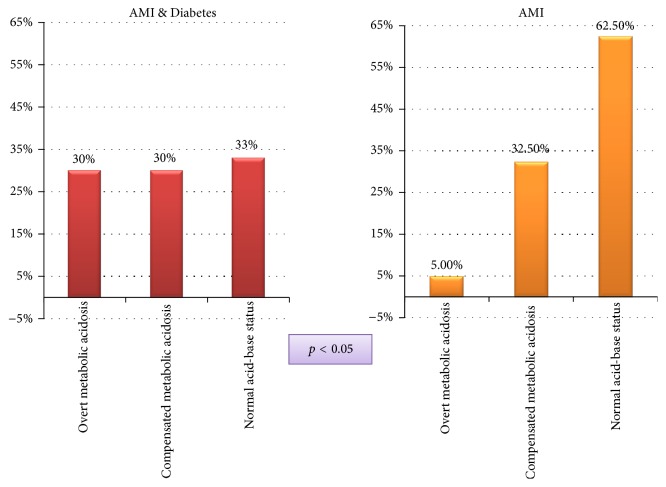
The distribution of acid-base disturbances among the patients with acute myocardial infarction and diabetes mellitus (AMI & Diabetes) and patients with acute myocardial infarction and normal glucose metabolism (AMI).

Patients with acute myocardial infarction and type 2 diabetes had a significantly lower mean pH value comparing to nondiabetics; they also had several times higher rate of metabolic acidosis (Figure 1). None of the cases could be attributed to respiratory acidosis; Spearman's rank correlation coefficients with bicarbonate levels were above 0.9, whereas correlation with PCO_2_ was nonsignificant; the individual analysis based on Davenport's diagram showed that all cases fall in the range of either normal acid-base status or compensated or overt metabolic acidosis.

Moreover, the mean partial carbon-dioxide pressures (PCO_2_) in the two groups of patients fell within the normal range (35.38 ± 8.72 mmHg and 36.09 ± 7.58 mmHg, resp.). Metabolic acidosis was related (*ρ* = 0.7, *p* < 0.05) to increased anion gap in the majority of cases. Impairment of acid-base balance in nondiabetics with AMI was strongly related to the incidence of heart failure and heart rhythm and conduction disturbances, as well as CK-MB isoenzyme level, partial oxygen pressure, PO_2_, and hemoglobin oxygen saturation, SO_2_, all of which indicating that the acidosis in this group of patients occurred primarily as the result of the increased lactate production. The correlation of pH values with lactate measurements was as high as *ρ* = 0.8, *p* < 0.001. In patients with AMI and type 2 diabetes the strongest and most significant correlations occurred between pH and SO_2_, CK-MB, lactate, heart rhythm and conduction disturbances, and blood ketones; the correlation between pH and SO_2_ prevailed the one between pH and plasma glucose (*ρ* = 0.68 versus *ρ* = 0.31, resp.) and the correlation between pH and lactate (*ρ* = 0.59) was stronger than the one between pH and blood ketones (*ρ* = 0.52). Only moderate correlation between pH and blood ketones along with nonsignificant correlation between pH and plasma glucose indicates that in these patients circulatory and oxygenation impairment caused by myocardial infarction may be more important cause of acid-base imbalance than diabetic ketoacidosis.

**Figure 2:**
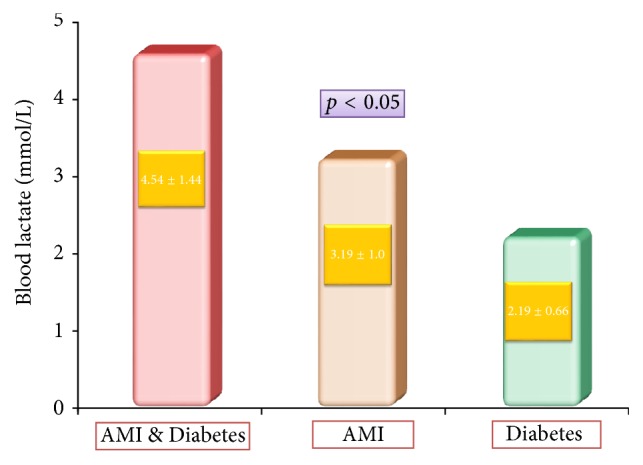
Mean blood lactate levels in patients with acute myocardial infarction and diabetes mellitus (AMI & Diabetes) and patients with acute myocardial infarction and normal glucose metabolism (AMI).

Furthermore, patients with AMI & Diabetes (Figure 2) had a significantly higher level of blood lactate than patients with AMI without diabetes (4.54 ± 1.44 versus 3.19 ± 1.005 mmol/L, *p* < 0.05, resp.); notably, mild hyperlactatemia was also registered in type 2 diabetes without cardiovascular, pulmonary, or renal impairment (Figure 2) and correlation coefficient between the lactate and plasma glucose levels in this group was *ρ* = 0.64. Apparently, both acute myocardial infarction and unregulated diabetes could contribute to lactate elevation in patients with AMI & Diabetes.

Lactate levels greater than 2 mmol/L represent hyperlactatemia, whereas lactic acidosis is generally defined as a serum lactate concentration >5 mmol/L. As much as 12.5% (5 patients) in AMI & Diabetes and 5% (2 patients) in AMI group had blood lactate levels in the range of lactic acidosis. While these numbers are too small for the reliable statistic discrimination, they still indicate that lactic acidosis might occur far more often in patients with diabetes suffering acute myocardial infarction.

In type 2 diabetic patients suffering myocardial infarction, blood lactate levels correlated with the occurrence of heart failure after AMI (*r* = 41) and heart rhythm and conduction disorders (*r* = 0.54), along with hemoglobin oxygen saturation (*r* = 0.68) and plasma glucose (*r* = 0.46). About one-third (e.g. 30%) of the patients with DM and AMI had usually mild to moderate level metabolic acidosis, while 12.5% had true lactic acidosis with lactate levels exceeding 5 mmol/L; of the latter, 75% suffered cardiogenic shock and/or pulmonary edema and 25% had fatal arrhythmias. Nondiabetics with AMI mostly had moderately increased blood lactate, but still these levels were significantly (*p* < 0.05) lower when compared to AMI & Diabetes group; here, lactic acidosis was registered in two cases (5%) and was always related to lethal outcome.

**Figure 3:**
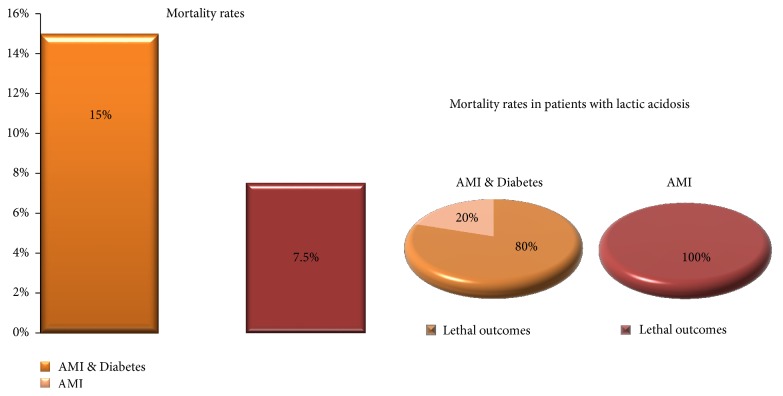
In-hospital mortality rate among the patients with acute myocardial infarction and diabetes mellitus (AMI & Diabetes) and patients with acute myocardial infarction and normal glucose metabolism (AMI) and prevalence of lethal outcomes among the patients with lactic acidosis in AMI & Diabetes and AMI groups.

Diabetics who experience acute myocardial infarction have worse clinical outcome comparing to patients without diabetes. In-hospital lethal outcomes in AMI & Diabetes group in our study were twice as high as those in nondiabetics (Figure 3). However, in the group of patients with diabetes and AMI, lactic acidosis was not always related to lethal outcome (Figure 3).

## 4. Discussion

Myocardial infarction can contribute to the increase in ketone bodies and lactate production by several mechanisms.

- Diabetic ketoacidosis is often triggered by myocardial infarction (MI) [8]. MI may precipitate hyperglycemia and DKA via an increase in counterregulatory hormones, such as epinephrine [9], both in type 1 and type 2 diabetes—mainly the subpopulation of type 2 diabetics with decreased insulin production suffering severe stress.
- Myocardial infarction can also contribute to the occurrence of hyperosmolar hyperglycemic state (HHS) in type 2 diabetics; although HHS itself may not directly cause intense acidosis, it may contribute to increased ketone and lactate production [10].
- Acute myocardial infarction is often related to impaired tissue oxygenation, arising either from decreased oxygen delivery or because of a defect in oxygen utilization [11] or inhibition of the mitochondrial pyruvate dehydrogenase complex; both mechanisms lead to increased anaerobic metabolism and increased production of lactic acid.

Lactic acidosis is the most common cause of metabolic acidosis in hospitalized patients [12]. Hyperlactatemia is commonly used as a diagnostic and prognostic tool in intensive care settings. While the prognostic role of hyperlactatemia in several critical ill diseases (such as sepsis and trauma) is well established, data in patients with acute cardiac conditions (i.e., acute coronary syndromes) are scarce and controversial. Blood lactate concentration obtained on emergency department on arrival may identify patients with critical cardiac illness (e.g., acute myocardial infarction), severe congestive heart failure, serious arrhythmias, and cardiogenic shock and correlates with a poor short-term outcome after AMI and cardiac surgery [13]. Lazzeri et al. [14], in the regression analysis, also proved that lactate levels were an independent predictor for intraintensive cardiac care unit mortality in patients with insulin resistance.

Several studies [15–22] suggested that even the lower lactate levels than those indicating overt lactic acidosis (e.g., >5 mmol/L) may be associated with the grave prognosis and that value depends on the specific disease [15–17, 19, 22]. The suggested lactate cutoff values ranged from as low as 0.75 mmol/L [20] up to 4 mmol/L [15, 16]. On the other hand, Lee at al. [23] outlined that only true lactic acidosis, not hyperlactatemia, was found to predict in-hospital mortality in critically ill patients with severe sepsis. Dynamic lactate measurements may be even better predictor of the outcome than the single initial preadmission or admission laboratory testing [20].

The correlation and regression analyses in independent studies [24–26] confirmed a prognostic value of blood lactate levels related to the outcome both in nondiabetic patients with insulin resistance and diabetic patients suffering acute myocardial infarction. However, the lactate cutoff value requiring intensification of treatment and additional care in order to avoid potentially lethal complications in these patients is not clear.

While the several recent studies focus on relationship between diabetic ketoacidosis and myocardial injury [27–29] the studies targeting both lactate production and the predictive value of lactate levels in diabetic population with MI are largely missing aside of those associated with metformin induced changes [30], despite the fact that myocardial infarction was well-recognized precipitating factor for the occurrence of lactic acidosis in people with diabetes [31].

It is obvious, however, that, by the result of our and other [32] studies, since the elevated lactate levels (2.19 ± 0.66 mmol/L) may be found in unregulated but otherwise uncomplicated diabetes, the cutoff values 0.75–3.5 mmol/L mentioned in the above studies would be hardly applicable to the diabetic patients with acute myocardial infarction. Furthermore, the determination of the lactate cutoff value may be not as simple as in nondiabetic patients, having in mind the number of factors influencing lactate levels. Some of those are unregulated diabetes stimulating adipose tissue [33] and kidney [34] to produce lactate; the action of stress hormones secreted in AMI may cause a mild to moderate increase in blood lactate level; tissue hypoxia leads to increased lactate production and delayed lactate clearance; systemic oxygen deficit and circulatory collapse increase the lactate production to the level typical for lactic acidosis; rarely, metformin therapy in combination with renal impairment may also contribute to hyperlactatemia. Apparently, all these processes may have a synergistic effect.

By the results of the correlation analysis performed in our study, measurement of lactate levels in diabetic patients following acute myocardial infarction may be useful in risk assessment regarding possible outcome and indicator of the need for treatment intensification in order to prevent complications—hyperosmolar prothrombotic state, hypoxia, heart rhythm or conduction disturbances, heart failure, pulmonary edema, cardiogenic shock, and possible lethal outcome. In nondiabetics suffering acute myocardial infarction the outcome of patients with lactic acidosis was inevitably fatal; in DM+AMI group all but one patient with lactic acidosis had a lethal outcome.

However, while the number of cases in the study by the results of power analysis was adequate for the estimation of the differences in blood lactate and PH levels (power of 0.8 and 1.0, resp.) between the groups, the number of patients with lactic acidosis (5 in AMI & Diabetes group, 2 in nondiabetics with AMI, power analysis <0.15) and the number of lethal outcomes (6 and 3, resp., power analysis <0.12) was insufficient for the reliable testing of the differences between the groups and, accordingly, for the risk assessment using logistic regression modeling. A precise determination of the cutoff value associated with the grave prognosis in diabetics suffering infarction, therefore, may be a task for the future studies focusing on serial lactate and the lactate clearance measurements and the estimation of dynamic lactate levels and trends over the treatment course.

## 5. Conclusion

Both defective glucose metabolism and low tissue oxygenation may contribute to the lactate production in diabetic patients with acute myocardial infarction; high lactate levels indicate increased risk for poor outcome in this population comparing to nondiabetic patients. The rise in blood lactate concentration in diabetics with AMI was associated with increased incidence of heart failure, severe arrhythmias, cardiogenic shock, and high mortality rate.

## Figures and Tables

**Table 1:** Basic demographic and clinical characteristics in three groups of patients.

	Diabetes + acute myocardial infarction	Acute myocardial infarction	Diabetes	*p*
Age	58.2 ± 11.7 yrs.	59.9 ± 13.2 yrs.	58.2 ± 17.4 yrs.	*p* > 0.05
Gender	m. 26 (65%)	m. 33 (82.5%)	m. 20 (50%)	*p* < 0.01^*∗*^
	f. 14 (35%)	f. 7 (17.5%)	20 (50%)	
Body mass index	27.41 ± 13.77 kg/m^2^	26.54 ± 14.25 kg/m^2^	24.38 ± 17.61 kg/m^2^	*p* > 0.05
Hypertensive	7 (17.5%)	5 (12.5%)	5 (12.5%)	*p* > 0.05
Postinfarct cardiac decompensation	17 (42.5%)	5 (12.5%)	/	*p* < 0.001
Cardiac rhythm disorders	18 (45.0%)	11 (27.5%)	/	*p* = 0.01
Cardiac conduction disorders	5 (12.5%)	4 (10.0%)	/	*p* > 0.05

^*∗*^Between the groups DM+AMI and AMI *p* = 0.07.
